# Airway Obstruction in Primary Care Patients: Need for Implementing Spirometry Use

**DOI:** 10.3390/diagnostics12112680

**Published:** 2022-11-03

**Authors:** Salvatore Bucchieri, Pietro Alfano, Palma Audino, Giovanni Fazio, Salvatore Marcantonio, Snamid Palermo Cooperative Group, Giuseppina Cuttitta

**Affiliations:** 1 Institute of Translational Pharmacology (IFT), National Research Council of Italy, Via Fosso del Cavaliere 100, 00133 Roma, Italy; 2 Triolo Zanca Clinic, Piazza Fonderia 23, 90133 Palermo, Italy; 3 Quality, Planning and Strategic Support Area, University of Palermo, Piazza Marina 61, 90133 Palermo, Italy; 4 Snamid Palermo Cooperative Group, Papa Pio X 18/20, 90142 Palermo, Italy

**Keywords:** airway obstruction, primary care, spirometry

## Abstract

(1) Background: To detect early airway obstruction in an adult primary care setting. (2) Methods: Seventeen general practitioners (GP) were involved. A total of 912 patients consulting their GPs over 40 years were recruited: 583 of them (323M) agreed to perform/undergo all the procedures: respiratory questionnaire, mMRC questionnaire, and spirometry. We identified four subgroups: physician COPD patients; physician asthma patients; asthma-COPD overlap syndrome patients; and no respiratory diagnosis subjects, on the basis of physician diagnosis. For screening purposes, an FEV_1_/FVC < 70% was considered a marker of airway obstruction (AO). (3) Results: Prevalence rates of COPD, A, and ACOS were 12.5%, 7.8%, and 3.6%, respectively. In the overall sample 16.3% showed airway obstruction: 26% mild, 56% moderate, 17% severe, and 1% very severe. In obstructed subjects, those reporting neither respiratory symptoms nor a physician’s respiratory diagnosis were 60% level I; 43% level II; 44% level III; and none level IV. Wheezing (*p* < 0.001), sputum (*p* = 0.01), older age (*p* < 0.0001), and male gender (*p* = 0.002) were the best predictors of airway obstruction. (4) Conclusions: A high prevalence of AO was found. In AO we found a high prevalence of subjects without respiratory symptoms or respiratory chronic diagnosis. Airway obstruction was predicted by the presence of wheezing, sputum, older age, and male gender.

## 1. Introduction

Chronic obstructive airway diseases, such as asthma or chronic obstructive pulmonary disease (COPD), are characterized by airflow obstruction (AO) and contribute significantly to morbidity and mortality worldwide and increase with life expectancy prolongation [1,2]. Hence airflow obstruction (AO) is a worldwide health problem with a significant impact on health status in the general population [1]. A recent longitudinal study showed an increasing trend in prevalence rates of all investigated respiratory symptoms and obstructive chronic diseases: in particular, current asthma attacks increased from 3.4% to 7.2% and COPD diagnosis increased from 2.1% to 6.8%, more than doubling in a period of 25 years, and asthma diagnosis prevalence increased, though not significantly, from 6.7% to 7.8%, [3]. An epidemiological study showed that in the general population with a smoking history, about 18% of subjects presented airflow obstruction, as assessed by spirometry [2]. The underdiagnosis of obstructive chronic diseases is a common problem, which affects the timing of therapeutic action, contributing to evolution toward more severe disease and impairing normal daily activities [4]. The World Health Organization (WHO) has long promoted an integrated approach toward better prevention, screening and treatment of all chronic airway diseases [5]. Spirometry is the cornerstone test that allows for the screening, diagnosis and monitoring of respiratory diseases. Appropriate testing may reduce the number of undetected cases as well as avoiding diagnostic misclassification [6]. An obstructive defect is indicated by a forced expiratory volume in one second/forced vital capacity (FEV_1_/FVC) ratio lower than 70% according to the ATS-GOLD definition. The Global Initiative for Obstructive Lung Disease (GOLD) recommends using the fixed ratio of FEV_1_/FVC < 0.70. The cut-off point chosen, 0.70, is based on the underlying assumption that this limit is a clinically useful marker of increased morbidity and mortality. An alternative approach is to define AO as FEV_1_/FVC less than the 5th percentile, the “lower limit of normal (LLN)” [7]. The use of one criterion rather than another has given rise to a controversial and still open debate in the literature. It seems that the age-related LLN of FEV_1_/FVC has been shown to reduce increases in COPD prevalence among the older healthy population when using the fixed-ratio criteria (FEV_1_/FVC < 0.7) and increase the diagnosis among younger patients [8]. Patients with chronic obstructive airway diseases, such as asthma and COPD, are associated with a worsening in quality of life, affective well-being, and an increase in the social and health burden of the disease [9,10,11]. In this context, primary care and GPs have a crucial role in prevention, screening, and management of chronic airway diseases [12]. From this perspective, the use of spirometry is crucial to achieving an early diagnosis, monitoring its evolution, and evaluating the efficacy of treatment [13,14]. Nevertheless, in primary care studies have highlighted critical aspects in the clinical and functional approach, such as insufficient expertise regarding institutional and international guidelines, time constraints in day-to-day clinical practice, failure in diagnosis, overlap between asthma and COPD regarding symptoms such as dyspnea, sputum and chronic cough, and poor familiarity with interpretation of spirometry parameters [15,16,17,18].

The aim of the present study was (1) to detect airway obstruction and severity using spirometry and (2) to evaluate the effect of multiple variables on obstruction in a primary care adult setting.

## 2. Materials and Methods

### 2.1. Study Design

**Figure 1:**
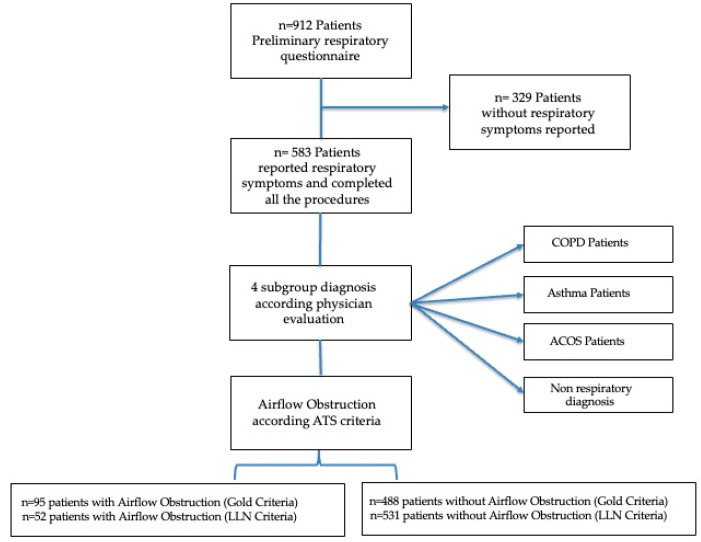
Patient flow diagram.

Seventeen general practitioners (GPs) (mean time since graduation was 30 years) participated in the study during January–June 2014: 912 patients, referred for a medical consultation (i.e., health problem, medical prescription, laboratory/instrumental test prescription) completed preliminary respiratory screening including age, smoking history, respiratory symptoms (cough, wheezing, sputum, dyspnea) and respiratory therapy. Only patients who reported respiratory symptoms participated in the study and completed all study procedures. A total of 583 patients who reported respiratory symptoms accepted and completed all the procedures (Figure 1). All 583 patients reported stable respiratory conditions in the last 6 weeks. The procedures of the study included a respiratory questionnaire (IMCA Indicators for Monitoring COPD and Asthma in the EU) [18], comorbidity evaluation, smoking habits, body mass index, respiratory symptoms, physician’s respiratory diagnosis, dyspnea perception using the modified Medical Research Council dyspnea scale (mMRC) [19], and spirometry. We identified 4 subgroups: physician COPD patients (COPD); physician Asthma patients (A); Asthma COPD Overlap Syndrome patients (ACOS); and non-respiratory diagnosis subjects (NRD), on the basis of the answers to the questions, the physician’s respiratory diagnosis and clinical evaluation. For screening purposes, a FEV_1_/FVC < 70% was considered a marker of airway obstruction [20] after post- bronchodilator (400 g of salbutamol) spirometry according to the American Thoracic Society criteria of acceptability and reproducibility. The threshold of the lower limit of normal (LLN) of FEV_1_/FVC [7] was also used for screening purposes.

Severity was determined by the FEV_1_ level: I mild (FEV_1_ ≥ 80% of the predicted value); II moderate (50–79% of the predicted value), III severe (30–49% of the predicted value); IV more severe (<30% of the predicted value). The study was approved by the Local Institutional Ethics Committee (N° 10/2013, 18 September 2013). All subjects gave written informed consent. The individual privacy of clinical data was guaranteed under Italian law.

### 2.2. Procedures

Dyspnea perception was evaluated by the Modified British Medical Research Council questionnaire (mMRC), a short questionnaire that allowed a numeric value to be placed on each subject’s exercise capacity. It is made up of a Likert scale describing the range of respiratory disability from 0 (none) to 4 (almost complete incapacity). In our study it was administered by an interviewer with the statements framed as questions. The score is the number that best fits the patient’s level of activity. mMRC is an instrument easily understood by patients and quick to fill in. Spirometry: Height and weight were measured in all patients in a standing position without shoes, using a stadiometer and an electronic digital scale: BMI was computed as weight/height^2^ (kg/m^2^). Oxygen saturation measurements were collected, before spirometry, using a wearable finger pulse oximeter. Pulmonary function tests were performed using a portable spirometer (MicroLoop, Micro Medical, Chatham Maritime, Kent, UK). Forced expiratory volume in one second (FEV_1_), forced vital capacity (FVC), and maximum mid-expiratory flow (FEF_25–75%_), on the curve flow-volume were measured according to the ATS/ERS guidelines [20], and the best FVC and FEV_1_ were retained. The predicted spirometric values were those proposed by the Global Lung Initiative (GLI). Comorbidities: The most common chronic comorbidities were metabolic disorders (i.e., hypertriglyceridemia, hypercholesterolemia), cardiovascular diseases (i.e., cardiopathy, atrial fibrillation, arrhythmia and IMA), hypertension, mental health disorders (i.e., depression, anxiety), respiratory diseases (i.e., COPD, asthma, OSAS, bronchiectasis), allergy, diseases of the musculoskeletal system (i.e., osteoporosis, arthropathy, low back pain), obesity, gastrointestinal disease (i.e., gastritis, colitis, hiatal hernia), thyroidopathy, other. The number of comorbidities was evaluated as the sum of all the comorbidities of each patient.

Data analysis: Statistical analyses were performed using SPSS version 20. The central tendency prevalence and measures were used to describe the anthropometric and clinical data. Physician diagnoses were compared with patient reports and spirometric categories. Associations with diagnosis were assessed with Fisher’s exact tests. Categorical variables such as cough, wheezing, sputum, dyspnea (mMRC > 1) differences and comparison of the distribution of interquartile range of age between the LLN criteria and FEV_1_/FVC < 70% fixed ratio were analyzed using chi-squared tests. Logistic multivariate regression analysis was performed to analyze the relationships between the study variables. *p* values < 0.05 were considered statistically significant.

## 3. Results

The anthropometric and clinical characteristics of the study sample, overall and separately for gender, are presented in Table 1.

**Figure 2:**
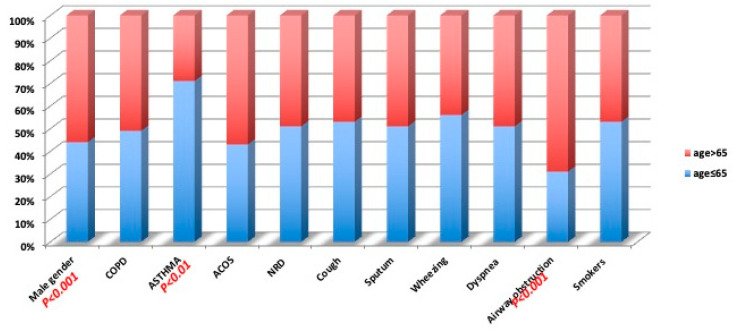
Prevalence of the studied variables for median age (65 years).

The study group included 583 subjects with a mean age (±SD) of 64 years (±10.80): 260 females (45%) and 323 males (55%); the mean age (±SD) of the female patients was 61.64 (±10.31) years and was significantly (*p* < 0.0001) lower than that of the males, 65.94 years (±10.82). In the overall sample, 191 (33%) subjects were current smokers, 237 (40%) former smokers, and 155 (27%) never smokers. Based on the respiratory health questionnaire, we found a prevalence rate of chronic cough of 27%, sputum 26%, wheezing 32%, and dyspnea 44%, in the overall sample. According to the physician’s respiratory diagnosis, we found a prevalence of 12.5%, for COPD patients, 7.8% for asthma patients, and 3.6% for ACOS patients. Table 2 shows the smoking history prevalence for reported physician diagnosis. The prevalence of gender, physician diagnosis, respiratory symptoms, airway obstruction, and smoking habits was evaluated for people younger than 65 years and people of 65 years or older, which is the median age of the overall sample (Figure 2). Figure 2 shows a significant prevalence of males among older participants (*p* < 0.001), of asthma diagnosis in younger participants (*p* < 0.01), and a significant prevalence of airway obstruction in older subjects compared with younger subjects (*p* < 0.001).

**Figure 3:**
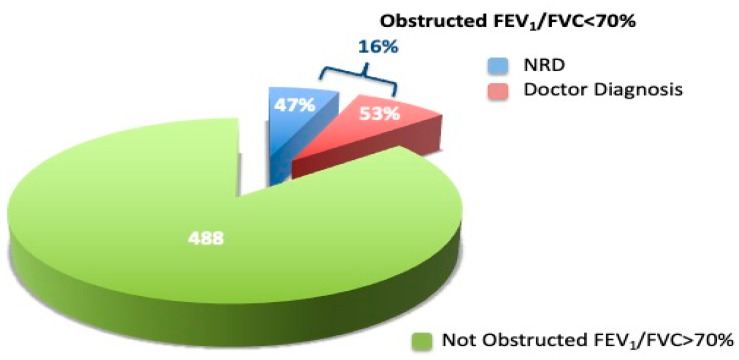
Airflow obstruction prevalence in the overall sample.

Airflow obstruction prevalence was found in 16.3% of the overall sample, according to the ATS criteria: 53% reported a physician’s respiratory diagnosis while 47% did not report a physician’s respiratory diagnosis (Figure 3). In the AO sample we evaluated the severity of the airflow obstruction on the basis of the FEV_1_ value, according to the ATS/ERS guidelines (ATS: statement), for each reported physician diagnosis subgroup (Table 3). Across them, 26% were classified as mild, 56% as moderate, 17% as severe, and 1% very severe.

Airflow obstruction prevalence was found in 9% of the overall sample, according to the LLN criteria. A significant difference was found between two criteria using chi-squared tests (*p* < 0.001). All subjects that showed obstruction with LLN were also detected with FEV_1_/FVC < 70% fixed ratio. When considering the remaining subjects detected only with fixed ratio and controlled by obstruction severity, we found the following obstruction severity: 44% were classified as mild, 49% as moderate, and 7% as severe.

The prevalence of smoking history and respiratory symptoms, such as cough, sputum, wheezing, and dyspnea in patients according to the two criteria was not significant. There are slight differences in age distribution between these two criteria as shown in Table 4 using chi-squared tests (*p* < 0.001) vs. (*p* = 0.02). What we have shown is that the GOLD criteria resulted in more patients being diagnosed as having AO.

Table 5 shows the prevalence of the studied variables for the sample with AO, separately for subjects with and without a physician reported respiratory diagnosis: 63% of the subjects with a diagnosis performed spirometry in the past versus only 17% of subjects without a diagnosis (*p* = 0.001). Obstructed subjects with a diagnosis also had more frequent sputum (*p* < 0.05) and wheezing (*p* < 0.05). We found a higher frequency of smokers (χ^2^ = 0.002) and a higher mMRC score (χ^2^ < 0.05) when comparing obstructed versus unobstructed subjects.

We found that 12% of obstructed subjects did not report respiratory symptoms (Table 6).

In a logistic regression analysis wheezing (*p* < 0.001), sputum (*p* = 0.01), older age (*p* < 0.0001) and male gender (*p* = 0.002) were the best predictors of airway obstruction (Table 7).

## 4. Discussion

In this real-life-observational study, we found that the prevalence of airflow obstruction in a sample of primary care adults was 16.3%. This result is consistent with an epidemiological study among the general Italian population, in which about 18% of subjects presented airway obstruction, as assessed by spirometry [2]. Similar results were reported with a further increase in AO in Italian, Norwegian and Swiss studies [21,22,23] although none of them are conclusive. The Italian research group suggested that tobacco use, air pollutants, urban living environment, and occupational exposures seemed to be the primary sources of an increase in AO. The Norwegian study reported a similarly strong increase in COPD prevalence from 1997 to 2005; however, it could be due to the methodological design of their study. For both the Italian and Norwegian studies, using follow-up data could have overestimated the increase in prevalence because of an aging study population. In a longitudinal study, West et al. [23] reported an increase in airway obstruction prevalence between 1993 and 2012 from 6.1% to 15.6% based both on GLI estimates and from 5.3% to 15.4% based on Hankinson estimates. Conversely, a Spanish research group reported a decrease in prevalence of COPD between the years 1997 and 2007, different from many other studies on temporal COPD trends. Their findings could potentially have been affected by a sicker population in 1997 than in 2007 [24].

The use of spirometry represents the gold standard for detecting airflow obstruction [25]. It could also help to investigate patients with respiratory symptoms and clarify the issues of differential diagnosis and confirming the diagnosis of chronic obstructive airway diseases in patients with respiratory symptoms. However, in general practice there is poor accessibility to spirometers and general practitioners are not always familiar with interpretation of the results [26]. In the current analyses, we plotted the respiratory diagnosis by the general practitioner against airflow obstruction and obstruction severity according to the ATS/ERS guidelines. Airflow obstruction prevalence varied among different criteria, such as FEV_1_/FVC < 70% fixed ratio, and LLN. In our study, a higher proportion of patients was detected with FEV_1_/FVC < 70% fixed ratio compared to LLN.

However, all subjects that showed obstruction with LLN FEV_1_.FVC were also detected with fixed ratio. The remaining obstructed subjects classified with FEV_1_/FVC < 70% showed an impaired FEV_1._ The differences in age distribution were slight between these two criteria. In a previous study the LLN were shown to underestimate lung volume [27].

These patients tend to be a relatively high age and to have an impaired lung function, so we suggest that in a primary care setting it is important to submit these patients to further clinical-diagnostic investigation in order to avoid the risk of misdiagnosis.

Chronic respiratory disease is common in primary care and coexists with other illnesses [28]. In the present study, a prevalence of 12.5% for COPD patients, 7.8% for Asthma patients and 3.6% for ACOS patients was found, in line with a previous study [21]. Checking by age, we found a higher prevalence of airflow obstruction in male subjects and in subjects aged > 65 years, in accordance with previous studies showing an effect of aging in an increase in respiratory diseases [29]. A further effect on increase in respiratory diseases seems to be played by different risk factors such as smoking, work exposure, air pollutants, low levels of education, and urban living [24,30]. In our study, we found nearly half of patients with airflow obstruction (47%) examined by a general practitioner did not receive any respiratory disease diagnosis. The remaining 53% showed airflow obstruction previously diagnosed by GPs. In agreement with Nardini et al. [15], we found that a correct diagnosis was more prevalent in subjects with previous spirometry or with respiratory symptoms. This result is consistent with recent studies reporting that a correct diagnosis is associated with previous spirometry [31,32]. Moreover, the Danish National Board of Health recommends that individuals >35 years, smokers/former smokers and/or relevant occupational exposure and at least one respiratory symptom should be offered spirometry to facilitate early detection of COPD [33].

As shown in the literature, an erroneous diagnosis of respiratory conditions may be common in primary care due to underuse or poor use of spirometry [28]. In our study, 51% of undiagnosed patients had a moderate obstruction, 33% had a mild obstruction and 15% had a severe obstruction. Among our patients with airflow obstruction 12% did not report respiratory symptoms as referred to in the IMCA questionnaire. So, the prevalence of airway obstruction, with a variable degree of severity, in patients without respiratory symptoms and without respiratory diagnosis, underlines the key role of spirometry and the role of scientific societies that should try to ensure that the general practitioner increases his or her familiarity with the use of spirometry and the interpretation of the results [16].

This could lead the GP to early detection of airway obstruction, the progression of pulmonary function decline, and the natural course of respiratory disease, as highlighted in Fletcher [34]. AO can be asymptomatic in its early stages and people who seem to have good self-reported health status and a lower comorbidity burden might be overlooked even when they have been exposed to risk factors (e.g., smoking). Early identification may have an essential role for global management of patients in terms of both benefit from therapies and decreasing exposure to risk factors so as not to miss opportunities to reduce the social and economic burden of the disease through early intervention strategies such as optimal management, smoking cessation support, and medication prescriptions. In fact, previous studies have shown that the burden of the disease increases when patients have already reached an advanced stage [35,36,37]. Different factors attributable to both the patient and the GP may delay diagnosis: on the one side, the patient has not communicated his/her symptoms to a general practitioner because of inadequate OD education regarding evaluation of his/her symptoms or because symptoms appear after the obstruction has occurred; on the other side, the general practitioner may fail to consider airway obstruction and, although the patient reports symptoms, may not prescribe spirometry [22]. In our study, respiratory symptoms seemed to help a physician take over. In particular, the presence of a clinical pattern such as wheezing, sputum, older age (>65 years) and male gender have been shown to predict airflow obstruction, whereas a less typical respiratory pattern and never having had a spirometry test were significantly associated with a lack of respiratory diagnosis. In the presence of symptoms such as wheezing, chronic cough, and sputum, spirometry should be used. Indeed, to think that such symptoms are only related to aging or smoking history [37,38,39] could lead to an underestimation of the clinical condition. In previous studies, contradictory results were reported on the role of age, gender, smoking history, and the clinical pattern in chronic airway diseases. Indeed, Hangaard et al. in their review found that misdiagnosis of COPD often occurs due to errors made in primary care [40], whereas Hill et al. [41] stated the absence of peculiar clinical characteristics in COPD diagnoses. Indeed, our findings suggest that men, rather than women, are at higher risk of AO underdiagnosis. Gender differences in symptom perception and attitudes toward medical care may contribute to these observations. In this connection, a previous study showed that women generally reported more dyspnea with a smaller airflow obstruction, and this is hypothesized to be due to a smaller inspiratory capacity related to a lower thoracic volume [42].

In conclusion, early detection of obstruction using spirometry in primary care is crucial for management strategies of the patient, with pharmacologic, non-pharmacologic, and preventive measures. It could help to reduce the burden of chronic airway diseases.

## 5. Limitations

The study has some limitations that must be considered in interpreting our findings. The leading limitation was linked to the definition of airflow obstruction. In the literature, the two main criteria used are FEV_1_/FVC < 0.70 proposed by the Global Initiative for Chronic Obstructive Lung Disease (ATS-GOLD) committee [43] and the lower limit of normal (LLN) according to the ERS definition [7,44]. Scientific activities and the GOLD guidelines have supported using fixed ratios as a consensus definition for airflow obstruction. However, some may argue that this simple ratio tends to over-diagnose the disease in older adults, as the fixed ratio is associated with an increased risk of death, whereas using the lower normal limit is not [45]. However, the wide confidence interval of LLN increases the likelihood of underdiagnosis of COPD in older individuals and in symptomatic smokers [46]. Previous studies investigated subjects in between the two definitions of airway obstruction and showed that their clinical profile is characterized by marked comorbidity and a poor health-related quality of life [47,48]. The literature contains a similar number of articles endorsing each of these competing definitions [49]. Moreover, the main purpose of the present study was to detect airway obstruction using spirometry in a primary adult care setting. A recent meta-analysis showed that individuals with airflow limitations, when evaluated with LLN or FEV_1_/FVC < 0.70 fixed-ratio criteria, show no significant difference regarding the risk of developing comorbidities [50]. According to Celli, the use of the fixed threshold of FEV_1_/FVC < 0.70 can be considered a useful, simple tool that has prognostic and practical value, thus helping to remove barriers to widespread use of spirometry [46,51]. In the present study, the sample was limited to patients living in an urban area of Sicily, which is a limitation when analyzing the results in the geopolitical context; therefore, generalization of the findings to other contexts should be approached with caution. During the screening we excluded patients that non reported respiratory symptoms, thus bias regarding the diagnostic procedure and the possibility to detect the accurate specificity and sensitivity of the method used. However, a previous study has demonstrated that the underdiagnosed subjects showed more symptoms, impaired ventilation and lung function decline through two-year longitudinal research compared with normal participants [52]. Moreover, we focused on subjects reporting at least one of the risk factors for obstructive airway disease. In addition, the use of the IMCA questionnaire, which is recognized for monitoring asthma and COPD and for description asthma and COPD prevalence and related symptoms, could reduce the bias and to lead towards a better assessment of patients

Another important aspect concerns the absence of socio-economic variables associated with our patients. Failure to adjust for these variables may have introduced a confounding bias. Some variables assessed in our study were based on self-reporting and thus a reporting bias (recall and social desirability) is possible. It is reasonable to suppose that those GPs who participated in the current study were more interested than many of their colleagues in management of respiratory conditions, thus excluding practices that usually used spirometry. Moreover, in this setting of the GPs body temperature were not appropriately studied.

## 6. Conclusions

The current study aimed to detect early airway obstruction in an adult population sample in a primary care setting using spirometry. We showed an elevated prevalence of airflow obstruction associated with different degrees of severity and without respiratory symptoms. Airflow obstruction was predicted by the presence of wheezing, sputum, older age, and male gender, indicating the need to implement spirometry in family practice as an early diagnosis tool. An accurate assessment and greater awareness of respiratory symptoms and lung function would help primary practitioners to improve management of patients with chronic airway diseases. The current results may encourage pulmonologists to collaborate with primary practitioners with the aim of improving the diagnosis.

## Figures and Tables

**Table 1:** Anthropometric and clinical characteristics of the study sample.

Sample	Overall n = 583	Females n = 260	Males n = 323	*p*-Values
Age (mean ± SD)	64 ± 10.80	61.65 ± 10.31	65.95 ± 10.83	*p* = 0.000
Gender, n (%)		260 (45%)	323 (55%)	
Body mass index (kg/m^2^) (mean ± SD)	27.9 ± 4.8	27.06 ± 4.94	28.57 ± 4.52	*p* = 0.000
Sp02 % (mean ± SD)	94 ± 2.6	94 ± 2.8	94 ± 2.5	*p* = 0.71
Smoking habits n (%)				*p* = 0.000
Current smokers	191 (33%)	87 (33%)	104 (32%)	
Non-smokers	155 (27%)	97 (38%)	58 (19%)	
Former smokers	237 (40%)	76 (29%)	161 (49%)	

**Table 2:** Prevalence of smoking history for each sub-group.

Diagnosis	Smoking History %	*p*-Values
COPD	98	*p* = 0.001
ASTHMA	51	*p* = 0.001
ACOS	76	NS
NRD	71	NS

COPD: chronic obstructive pulmonary disease; ACOS: asthma COPD overlap syndrome; NRD: non-respiratory diagnosis.

**Table 3:** Obstruction severity prevalence for each reported physician diagnosis.

	COPD	Asthma	ACOS	NRD
AO Stage	%	%	%	%
I	4	5	1	16
II	22	6	4	24
III	4	1	4	7
IV	1	0	0	0

COPD: chronic obstructive pulmonary disease; ACOS: asthma COPD overlap syndrome; NRD: non-respiratory diagnosis. The percentages are based on the total number of subjects with the diagnosis.

**Table 4:** Obstruction severity prevalence for two diagnostic criteria by interquartile range.

Interquartile Range (Years)	Diagnostic Criteria (FEV_1_/FVC < 0.7) ^§^ (% Patients)	Diagnostic Criteria (FEV_1_/FVC < LLN) * (% Patients)
39–57	10.5	19.2
58–64	18.9	21.2
65–72	28.4	17.3
73–92	42.1	42.3
*p*-values	0.001	0.022

The data showed the percentage of patients with airflow obstruction. * Post-bronchodilator FEV_1_/FVC < lower limit of normal was used for air flow limitation classification; ^§^, postbronchodilator FEV_1_/FVC < 70% was used to define air flow limitation according to the GOLD criteria; comparison of the distributions of age by interquartile range between the LLN criteria and FEV_1_/FVC < 0.70 fixed ratio were analyzed using chi-squared tests.

**Table 5:** Obstruction severity prevalence for each physician diagnosis subgroups.

Studied Variables	AO without Diagnosis %	AO with Diagnosis %	*p*
Male gender	37	35	NS
Age > 65	34	36	NS
Smokers	41	45	NS
Pack years	27	27	NS
Spiro ever	17	63	*p* = 0.000
Cough	18	27	NS
Sputum	18	23	*p* < 0.05
Wheezing	25	38	*p* < 0.05
Dyspnea	26	38	NS
Symptoms presence	39	48	NS

**Table 6:** Prevalence of studied variables for AO with and without symptoms.

	Symptom	
Airflow Obstruction Stage	Yes	No
I	27%	45%
II	52%	45%
III	19%	9%
IV	2%	0%
Tot AO subjects	88%	12%

**Table 7:** Logistic multivariate model to evaluate the effect of studied variables on obstruction.

Studied Variables	O.R.	95% Lower	95% Upper	*p*-Value χ²
Cough	1.27	0.70	2.28	NS
Sputum	2.11	1.15	3.62	0.01
Wheezing	3.50	2.06	6.32	<0.001
Dyspnea	1.38	0.79	2.39	NS
Age (>64 yrs.)	2.79	1.69	4.97	<0.001
Gender (M)	2.64	1.36	4.25	0.00
Smoking habits	1.82	0.912	3.64	NS
Comorbidities Number	1.53	0.921	2.551	NS
Cardiovascular	1.55	0.757	3.193	NS
Obesity	0.47	0.254	0.893	0.02

## Data Availability

Primary data are available upon request.
